# Cost and Cost-Effectiveness Evidence for Hospital-at-Home Programs in Asia: A Systematic Review

**DOI:** 10.3390/healthcare14213654

**Published:** 2026-10-28

**Authors:** Liang Guo, Jia Hui Chai, Luming Shi

**Affiliations:** 1 Singapore Clinical Research Institute, Consortium for Clinical Research and Innovation, Singapore 139234, Singapore; liang.guo@scri.cris.sg (L.G.); jiahui.chai@scri.cris.sg (J.H.C.); 2 Cochrane Singapore, Singapore 139234, Singapore; 3 Duke-NUS Medical School, Singapore 169857, Singapore

**Keywords:** hospital-at-home, home hospitalization, home-based care, health services research, Asia, cost-effectiveness, economic evaluation, healthcare costs, length of stay, systematic review

## Abstract

**Highlights:**

**What are the main findings?**

- This systematic review identified only five eligible studies from four Asian countries/regions, highlighting the limited availability of economic evidence on Hospital-at-Home (HaH) programs in Asia;
- Four of five studies reported cost savings alongside favorable findings for selected clinical outcomes, although the outcomes assessed and magnitude of effects varied across studies.

**What are the implications of the main findings?**

- Available evidence suggests that Hospital-at-Home programs may reduce healthcare costs in some Asian healthcare settings while maintaining comparable or improved outcomes; however, the evidence remains limited and heterogeneous;
- More rigorous economic evaluations incorporating quality-of-life outcomes and formal cost-effectiveness modeling are needed to support policy implementation and scaling decisions.

**Abstract:**

**Background/Objectives**: Hospital-at-home (HaH) programs are increasingly adopted as an alternative to inpatient care and have demonstrated cost savings and comparable clinical outcomes in Western healthcare systems. In Asia, where healthcare systems face growing hospital-bed capacity constraints, evidence on the economic impact of HaH remains limited. This systematic review aimed to appraise and synthesize evidence on costs, resource utilization, and formal cost-effectiveness evaluation, where available, of HaH programs for patients eligible for general hospital admission within Asian healthcare systems. **Methods**: This systematic review was conducted according to PRISMA guidelines and registered in PROSPERO (CRD420251117897). Literature searches were performed in PubMed/MEDLINE, EMBASE, and the Cochrane Library from 2000 to June 2026. Studies evaluating HaH or similar home-based hospitalization models in Asian settings and reporting cost-related outcomes were included. Methodological quality was assessed using the Joanna Briggs Institute checklist for economic evaluations, and findings were synthesized using a dominance ranking matrix. **Results**: Of 2243 records identified, five studies from four Asian countries/regions involving 10,152 patients met the inclusion criteria. Four studies reported cost savings alongside favorable findings for selected clinical outcomes, including shorter hospital stays, although findings varied across outcomes and settings. No study reported a formal cost-effectiveness analysis. Overall, the dominance ranking matrix favored HaH in most scenarios, although one study reported less favorable outcomes. **Conclusions**: The limited available evidence suggested that HaH programs may reduce healthcare costs alongside favorable findings for selected clinical outcomes in some Asian healthcare settings. However, substantial heterogeneity and the absence of formal cost-effectiveness evaluations limit definitive conclusions regarding their cost-effectiveness.

## 1. Introduction

Hospital-at-home (HaH) has evolved over the past three decades as a viable alternative to traditional inpatient hospitalization, offering acute care services in the patient’s home through coordinated, multidisciplinary teams. This model addresses growing healthcare demands on healthcare systems by reducing reliance on hospital beds while maintaining clinical standards. Evidence from North America, Europe and Australia have shown that HaH reduces costs by 19% to 30% per episode while achieving comparable clinical outcomes and high levels of patient satisfaction [1,2,3]. These findings have established HaH as a cornerstone of value-based care in many advanced health systems.

In Asia, health systems face significant capacity issues due to high population density, limited hospital infrastructure, and increasing demand for chronic and acute care services. These pressures have accelerated interest in HaH programs across countries such as Malaysia [4], Singapore [5], China [6], and Japan [7] to address these challenges. It is important to establish not only clinical equivalence but also cost savings, particularly given the diverse healthcare financing models in Asia. Although numerous systematic reviews have examined the cost-effectiveness of HaH in Western settings, evidence from Asian contexts remains scarce and fragmented.

Understanding the economic implications of HaH in Asia is critical for policymakers and healthcare providers seeking sustainable solutions to hospital overcrowding and rising costs. This systematic review aims to critically appraise and summarize current evidence on costs, resource utilization, and formal cost-effectiveness evaluation, where applicable, of HaH programs for patients eligible for general hospital admission within Asian healthcare systems. By consolidating the available data, this review seeks to inform future research and guide policy decisions on scaling HaH models in the region.

## 2. Materials and Methods

This systematic review has been registered in the international prospective register of systematic reviews (PROSPERO) under the registration number: CRD420251117897. The Preferred Reporting Items for Systematic Reviews and Meta-Analyses (PRISMA) guidelines were adopted to identify eligible studies.

### 2.1. PICO Elements

- Population: Patients who require hospital admission in an emergency department or general ward in a general hospital;
- Intervention: Hospital-at-Home (HaH), or “medical at home” program;
- Control: Traditional ward-based care;
- Outcome: Formal economic evaluation, including cost-effectiveness analysis (CEA) in terms of incremental cost-effectiveness ratio (ICER), cost–utility analysis (CUA), cost–benefit analysis (CBA), and cost-minimization analysis (CMA) where applicable. In the absence of formal economic evaluation, studies reporting both relevant clinical outcomes (e.g., length of stay, readmission, or mortality) and cost outcomes were considered.

### 2.2. Search Strategy and Eligibility Criteria

A literature search was performed in PubMed/MEDLINE, EMBASE, and the Cochrane library, from 2000 to Jun 2026 with language restriction of English only. Search terms included were “hospital at home”, “HaH”, “medical home”, “home care service”, “cost analysis”, “cost effectiveness”, “economic evaluation”, “length of stay”, “readmission”, and related synonyms. The search included both MeSH terms and free text. In addition, all reference lists in the included articles and relevant review articles were manually checked. The search strategy for the 3 databases available is attached in the Appendix A.

Studies were included if they matched our predefined PICOs and provided cost analysis data. We included primary research studies of any study design that evaluated the costs and/or economic outcomes of HaH interventions and provided relevant cost data. Studies were excluded if “home care” were delivered in settings such as nursing homes, home births, palliative services, hemodialysis, rehabilitation, chronic disease management, primary care, health education, or nutrition/exercise interventions, as there were outside the scope of this review. Study protocols, other systematic reviews, and meta-analysis were also excluded.

All records identified from the literature searches across the three databases were imported into Covidence for citation management and study selection. Two reviewers (GL, JH) independently screened the titles and abstracts for the first most relevant 100 records, according to Covidence most relevant rating, as a calibration exercise to ensure consistent interpretation and application of the predefined eligibility criteria. Discrepancies were discussed and resolved by consensus before one reviewer completed the remaining title and abstract screening. Full-text eligibility assessment was subsequently conducted independently by two reviewers (GL, JH). Any disagreements at this stage were resolved through discussion and consensus. Where consensus could not be reached, a third reviewer (LM) was consulted to make the final decision.

### 2.3. Data Extraction

A predefined data extraction template was set up before the literature screening started. Baseline characteristics consisted of the first author, year of publication, study design, total number of participants enrolled, gender and age, and study setting. All relevant data for comparative economic evaluations were extracted including effectiveness and cost analysis related information consisting of economic perspective, time horizon, main effectiveness outcomes, cost estimates, data source, cost type, currency, sensitivity analysis, and any co-variate considered for adjusted model. Data extraction was performed independently by two reviewers, with results cross-checked upon completion. Any discrepancies were resolved by a third reviewer.

### 2.4. Assessment of Quality and Risk of Bias

The methodological quality of included economic evaluations was assessed using the Joanna Briggs Institute (JBI) Critical Appraisal Checklist for Economic Evaluations [8]. Each item on the checklist was evaluated and recorded as “Yes”, “No”, “Unclear”, or “Not applicable”. Consistent with JBI guidance, the appraisal focused on the methodological rigor and transparency of each study. For descriptive purposes, the proportion of applicable JBI criteria rated “Yes” was calculated for each study, with items rated “Not applicable” excluded from the denominator. These percentages were used to facilitate comparison across studies rather than as validated JBI quality scores. Methodological quality was primarily interpreted based on the item-level judgments. The assessment was independently conducted by two reviewers, and any disagreement resolved by a third reviewer.

### 2.5. Analysis

Clinical outcomes and cost data were synthesized separately before an integrated assessment of the direction of clinical and economic findings. A meta-analysis was considered where sufficiently comparable data were available; however, owing to heterogeneity in the included studies and outcome measures, quantitative pooling was not considered appropriate. Clinical outcomes and cost data were, therefore, synthesized narratively and reported separately where comparative analyses were unavailable. As none of the included studies conducted a formal cost-effectiveness analysis, ICERs or other summary measures of cost-effectiveness were unavailable for quantitative synthesis.

To synthesize the direction of cost and health outcomes across the included studies, the dominance ranking matrix (DRM) [8], developed by the Joanna Briggs Institute (JBI), was utilized. The DRM is a classification tool that interprets economic evaluation results in systematic reviews, presenting the analysis as a permutation matrix with nine possible cost-effectiveness outcomes. In this review, a study was categorized as “health benefit+” if improvement was observed in any of the main clinical outcomes, such as length of stay, readmission, or mortality; “health benefit−” indicated poorer clinical outcomes, while “health benefit 0” indicated no meaningful difference among groups. Similarly, “cost−” indicated lower costs or cost savings associated with the intervention, “cost+” indicated higher costs, and “cost 0” indicated no meaningful difference in costs. These classifications were based on the direction of the reported comparative findings rather than on results of a formal economic evaluation. The corresponding combination of cost and health outcomes was then mapped to the nine cells of the DRM and classified as favor intervention, rejection, or unclear. The overall distribution of studies across these classifications was then used to provide an integrated, directional interpretation of the reported clinical and cost outcomes.

## 3. Results

### 3.1. Characteristics of the Studies

**Figure 1:**
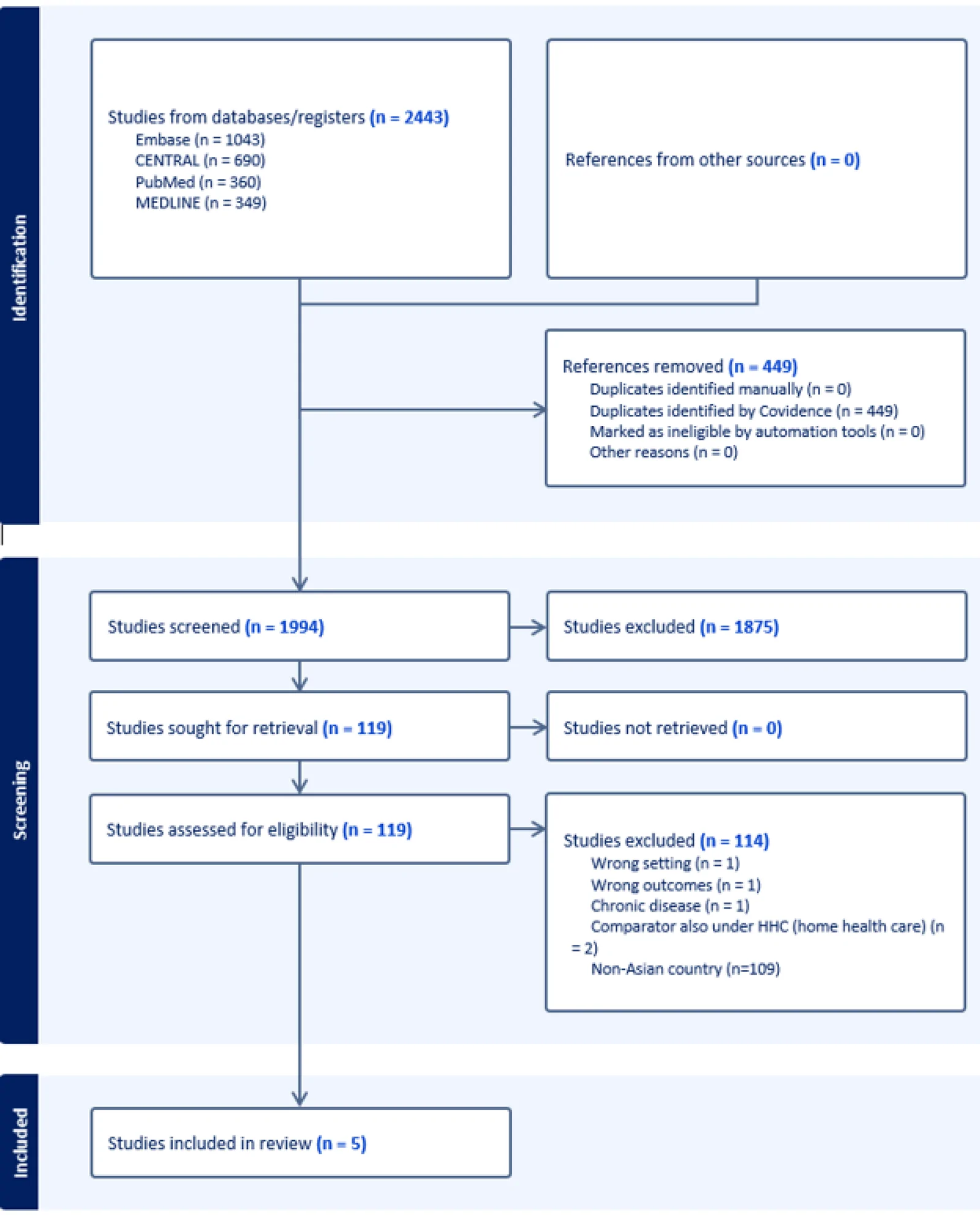
PRISMA flow diagram.

A total of 2443 articles were identified in our literature search among PubMed/MEDLINE, EMBASE, and the Cochrane library, based on the specified search criteria. One hundred and nineteen eligible full-text articles and abstracts were assessed after removal of duplications and initial screening. According to the inclusion criteria, five studies [9,10,11,12,13] were ultimately selected for the systematic review (Figure 1).

Table 1 summarized the main characteristics of the included studies. The studies originated from four different countries or regions, of which three were retrospective or cross-sectional observational studies and two were quasi-experimental studies. Three of the papers are published recently in 2025 and 2026, while one in 2019 and another in 2007. In total, 10,152 eligible patients were included in the analysis, with average ages ranged from 51.4 to 81.9 years. Most studies focused on elderly population. The home healthcare models reported in the five studies varied to some extent, though all fell within the categories of home hospitalization (HH), transitional care (TC) model, hospital-at-home (HaH), or home healthcare services (HHS). While the main functions of these care models differ slightly, all involve multidisciplinary teams with wide range of specialists caring for patients. Additionally, the inclusion criteria varied across studies. The Isreal study was a government-led approach in which elderly were widely included in HaH based on the medical condition. The Turky study focused on patients from the emergency department, while the other studies recruited patients from general inpatient settings.

Quality assessment of all the included studies was conducted using JBI Critical Appraisal Checklist for Economic Evaluations. Item 7, “Are costs and outcomes adjusted for differential timing?” was not applicable to any of the studies, as none had performed formal economic evaluations requiring such adjustment. Consequently, this item was excluded from the denominator when calculating the descriptive proportion of applicable criteria rated “Yes”. For the same reason, all the studies were marked as “No” for the criterion “Has clinical effectiveness been established?”. Studies were also penalized if there was no incremental analysis or sensitivity analysis conducted.

The proportion of applicable criteria rated “Yes” ranged from 40% to 90% across the included studies. Ang and Ko met 80% and 90% of applicable criteria, respectively, while Jacobs met 70% and Tan and Ayyildiz each met 40%. Item-level judgments are presented in Table 2 to facilitate interpretation of specific methodological strengths and limitations.

### 3.2. Key Findings

Table 3 presents results of all the studies. None of the included studies conducted formal cost-effectiveness analysis or reported an ICER. The 2 Singapore studies performed comparative analysis for adjusted differences among groups, controlling for relevant covariates. These studies adopted different perspectives: Ang considered both patient and government viewpoints, while Ko at focused on healthcare system perspective, collecting costs across all related activities. Both studies reported significantly shorter lengths of hospital stay (by 3 or 4.2 days) and lower healthcare costs compared with their respective comparator groups. Ang observed fewer hospital readmissions, emergency facility visits, and higher mortality rates, though these findings were not statistically significant. Ko noted a higher readmission at 30 days in HaH group, but this was also not statistically significant due to a wide confidence interval. Ko additionally reported quality-of-life data, with no observed difference among groups. Ko’s study also conducted sensitivity analysis that confirmed the robustness of their base-case results.

Studies from Taiwan and Turkey reported clinical outcomes and cost data across different patient groups, enabling meaningful comparisons. Tan observed a positive impact of the HaH model, with a significant shorter length of stay (LoS) and reduced costs; however, other clinical parameters did not demonstrate significant advantages. Patient satisfaction was also assessed, and all respondents described the program as “satisfied”. In contrast, Ayyildiz reached an entirely different conclusion, reporting longer LoS in both ward and ICU, lower survival rates, and higher cost for the intervention group, all statistical significance.

Jacobs’s study in Isreal offered a unique perspective by evaluating changes after the HaH program was phased out. Following the government’s discontinuation of the program, there was a marked increase in hospital visits and healthcare expenditures among previously enrolled patients. Although patient-level data were not available, the overall increase trend showed significant rises in hospitalization, recurrent admissions, and costs. The ratio of increased costs to prior savings was 5:1, supporting the hypothesis that discontinuing HaH would lead to higher hospital utilization rates among the local elderly population.

### 3.3. Synthesis of the Studies

Table 4 presented the dominance ranking matrix for the reviewed studies. It indicates that four out of five studies were classified within the “favor intervention” domain of DRM, reflecting concurrent improvement in selected clinical outcomes and cost savings. However, one study (Ayyildiz) with higher cost and lower health benefit led to “reject intervention” domain. Taken together, while most of the evidence favors HaH, variability across context should be noted and the potential for adverse outcomes in certain settings. Overall, these findings should be interpreted as a directional synthesis of clinical and cost outcomes rather than evidence from formal cost-effectiveness evaluations.

## 4. Discussion

With the current policy emphasis on “care closer to home” [14] (WHO 2021 to 2030), and growing concern about the rising demand for hospital-bed-based care, research on economic impact of the home-based care has become increasingly important. Over the past two decades, numerous systematic reviews have examined this topic [15,16,17], most notably the Cochrane Review initiated by Shepperd and colleagues on early discharge Hospital-at-Home (HaH), which has been updated multiple times and provides broad coverage of the available evidence [18]. However, these reviews predominantly include studies from Western countries with more developed healthcare systems, leaving a significant gap in evidence in Asia. This gap likely reflects the scarcity of comprehensive and rigorous economic evaluations conducted in Asian contexts, a finding consistent with the observations of this review. During the literature screening process, 109 potentially relevant studies reporting economic evidence were excluded because they were conducted outside Asia. This underscores an urgent need for high-quality research focused on the economic impact of HaH programs within Asian healthcare systems.

This review identified five studies analyzing costs associated with HaH and similar programs across four Asian countries and regions. None of the included studies reported formal cost-effectiveness evaluations. Nevertheless, comparative analyses of clinical and cost outcomes provided preliminary evidence that HaH may be associated with cost savings alongside comparable or favorable findings for selected clinical outcomes in some settings. Findings varied across individual outcomes, particularly for readmission and mortality, and should, therefore, not be interpreted as demonstrating an overall improvement in clinical outcomes. Considerable variation was also observed in program design, patient populations, cost perspectives, time horizons, and healthcare system contexts.

Since none of the included studies reported formal cost-effectiveness evaluations, items requiring detailed economic methods (e.g., disaggregated direct and indirect costs, modeling approaches, discounting procedures, or cost-effectiveness acceptability curves) were not applicable for data extraction. Furthermore, the studies varied in healthcare system settings, patients’ inclusion criteria, and HaH program designs, as well as in analytical perspectives. These variations rendered quantitative pooling of effectiveness data unfeasible. In this context, a three-by-three dominance ranking matrix (DRM) was utilized to classify each study’s overall economic finding (favored/unclear/rejected). The DRM—recommended in the JBI guidance and other methodological papers—provides a pragmatic, visual approach to summarizing heterogeneous or partial economic evidence when quantitative pooling synthesis is not feasible and has increasingly been used in recent systematic reviews of economic studies [19,20,21]. The matrix provided a structured approach of synthesizing the direction of cost and clinical outcomes and indicated that four of the five included studies fell within the “favor intervention” category. However, the DRM should, therefore, be interpreted as a directional synthesis of the reported cost and clinical outcomes rather than as evidence of formal cost-effectiveness. In addition, the classification does not fully account for differences in study design, methodological quality, population, magnitude or precision of effect estimates, or healthcare context across studies.

Regarding the populations studied, the five included studies focused on the elderly, which may limit the generalizability of HaH program effectiveness to the broader population. Furthermore, Ayyildiz was the only study reporting adverse findings, with both clinical outcomes and cost data suggesting rejection of the intervention. This may be attributable to the characteristics of the intervention group, which was significantly older and included more females compared with the control group. Older age is typically associated with higher healthcare expenditures, while female sex may also be linked to elevated costs [22], although evidence on gender differences remains inconsistent. The baseline differences between the intervention and comparator groups, particularly in age, may, therefore, have contributed to residual confounding and influenced the observed cost differences. Additionally, several methodological limitations identified through the item-level JBI appraisal may further reduce confidence in these findings.

HaH programs are also associated with increased patient satisfaction and potential economic benefits, supporting continued evaluation of these models as alternatives to conventional inpatient care [23,24]. However, QoL data were reported in only one included study (Ko et al.). In contemporary health economic evaluations, QoL outcomes have become increasingly important alongside traditional clinical and cost measures [25], particularly in home-care services, where the primary objective is to enhance patient comfort, satisfaction, and overall quality of life [26]. Incorporating QoL allows for a more comprehensive assessment of healthcare interventions, especially in chronic or non-fatal conditions where survival alone may not capture patient-relevant benefits [27]. Measures such as quality-adjusted life years (QALYs) and patient-reported outcome instruments enable the integration of both length and quality of life into economic models, facilitating comparisons across interventions and informing resource allocation decisions. Consequently, consideration of QoL outcomes enhances the relevance and patient-centeredness of economic evaluations, supporting policymakers in prioritizing interventions that improve both longevity and well-being.

Regarding quality assessment, this review utilized the JBI Critical Appraisal Checklist for Economic Evaluations, a widely recognized tool for assessing the methodological quality of health economic evaluation studies. However, the checklist does not contain a specific item evaluating the appropriateness or justification of the chosen evaluation perspective, with this aspect only indirectly addressed under the criterion of whether a well-defined review question is stated. This has been highlighted as a limitation when compared with other economic evaluation appraisal tools [28], such as the Drummond checklist and the Consensus on Health Economic Criteria (CHEC) checklist. Although the original protocol proposed the use of the CHEC checklist, no included studies reported a full cost-effectiveness analysis, rendering several CHEC items inapplicable. The JBI checklist was, therefore, selected as a more applicable alternative for the available evidence, while its limitations for assessing some aspects of economic evaluation, including analytical perspective, are acknowledged [29,30].

A formal assessment of reporting bias was not conducted due to the limited number of included studies and the absence of quantitative meta-analysis. Similarly, a formal certainty-of-evidence assessment following the GRADE approach was not undertaken, this should be considered when interpreting the strength of the overall evidence.

This review has several limitations. First, this review excluded research focused exclusively on end-of-life care, dialysis, rehabilitation, or tele-care model. While this approach ensured relevance to the scope of HaH programs examined in this review, it may have excluded studies reporting comprehensive cost-effectiveness data for specific populations or settings. Economic evaluations may also vary across countries because of differences in healthcare systems and reimbursement structures, and these variations are amplified for patients with diverse diagnosis and comorbidities. Importantly, none of the included studies conducted a formal cost-effectiveness analysis; therefore, the observed combination of cost savings and favorable clinical outcomes should not be interpreted as establishing the cost-effectiveness of HaH. Furthermore, the included interventions represented heterogeneous models of home-based care that differed in their organization, intensity, target populations, and healthcare settings. The findings should, therefore, not be interpreted as reflecting the effectiveness or economic impact of a single standardized HaH model, and their applicability to other HaH programs may be limited. Future research or systematic reviews may benefit from focusing on specific disease groups or clinical populations to improve the precision and applicability of the findings.

## 5. Conclusions

Despite methodological limitations and the absence of formal economic evaluations, synthesis of the available clinical and cost outcomes provides preliminary evidence regarding the potential economic implications of HaH programs in Asian healthcare settings. The limited available evidence suggests that these models may be associated with favorable findings for selected clinical outcomes alongside cost savings in some settings. However, these findings should not be interpreted as establishing the cost-effectiveness of HaH, given the small and heterogeneous evidence base and absence of formal cost-effectiveness analyses. Future studies should adopt rigorous economic evaluation frameworks, incorporate patient-centered outcomes, and investigate clearly defined populations and HaH models within specific healthcare contexts to better inform policy and implementation decisions.

## Figures and Tables

**Table 1:** Characteristics of included studies.

	Jacobs 2007 [11]	Ang 2019 [9]	Tan 2025 [12]	Ayyildiz 2025 [10]	Ko 2026 [13]
**Year**	2007	2019	2025	2025	2026
**Country/Region**	Israel	Singapore	Taiwan	Turkey	Singapore
**Paper Type**	Journal publication	Letter to editor	Journal publication	Journal publication	Journal publication
**Study Design**	Natural experiment descriptive	Retrospective	Retrospective cohort	Cross-sectional observational	Prospective quasi-experimental
**Study Period**	Jan 2000 to Oct 2003	April 2012 to March 2014	Aug 2024 to Jan 2025	Jan 2021 to Dec 2022	Jan 2021 to May 2023
**Settings**	Whole healthcare system in Jerusalem area	Tertiary hospital	Tertiary medical center	Emergency department of a tertiary care facility	One tertiary hospital and one general hospital
**Care Model**	Home hospitalization (HH)	Transitional care (TC) model	Hospital-at-home (HaH)	Home healthcare services (HHS)	Hospital-at-home (HaH)
**Sample Size for Analysis**	250 (ceased HH)	Total: 695 TC: 533 non-TC: 162	Total: 315 HaH: 69 Control: 246	Total: 8590 HHS: 866 non-HHS: 7724	Total: 302 HaH: 151 Control: 151
**Patient Age, mean (SD)**	73	TC: 81.9 (10.0) non-TC: 80.1 (12.0) *p* = 0.046	HaH: 79.9 (9.9) 78.9 (12.7)	76.4 (7.44) HHS: 78.80 (7.36) ^1^ non-HHS: 76.10 (7.40)	HaH: 58.0 (20.9) Control: 51.4 (21.2)
**Female, *n* (%)**	/	TC: 35.1 non-TC: 32.7	HaH: 48 (69.6) Control: 170 (69.1)	4145 (48.3) HHS: 461 (53.2) ^1^ non-HHS: 3684 (47.7)	HaH: 75 (49.3) Control: 68 (45.0)
**Economic Perspective**	Healthcare system	Patient and government	/	Healthcare system	Healthcare system
**Healthcare System Setting**	Universal multi-payer healthcare system, co-payment	Multi-layered health insurance system, co-payment	Single-payer universal health insurance system, co-payment	Universal health coverage with shared financing system, co-payment	Multi-layered health insurance system, co-payment
**Inclusion Criteria**	Elderly general medical patients at the acute or sub-acute stages of illness, palliative end of life care, and home rehabilitation, with illness that would otherwise require hospitalization and be functionally homebound under HH model.	Meet one of the criteria: 1. Older adult with dementia, frequent falls, functional decline post-hospitalization or urinary/bowel incontinence 2. Complex medical: COPD, CHF or CKD with ≥3 admissions within 1 year; poorly controlled diabetes; acute stroke with new disability; thromboembolism that requires anticoagulation 3. Complex nursing needs with stage 3 or 4 pressure sores, infected wounds, stoma care or IUT 4. Socially at risk individuals with inadequate care or need caregiver training	HaH patients diagnosed with pneumonia, urinary tract infection, or soft tissue infection	Aged 65 years and older who presented to the ED and visited at least twice per calendar month by hospital’s home health unit	Clinically stable, able to self-care or manage with caregivers, lived within the program’s catchment area and agreed to HaH admission
**Control Group**	HH model ceased	Patients who declined TC	Selected based on age, sex, and diagnosis	Patients do not receive HHS	Eligible but outside the catchment area or due to full capacity
**Exclusion Criteria**	/	1. Discharged to institutional care 2. End-of-life patients 3. Currently receiving home medical and nursing services 4. Readmitted or died within 7 days post-discharge	Rehospitalized during HaH course and those with insufficient data in the comparison cohort	Aged 18 to 64 years; Trauma patients	Patients who declined HaH
**Main Disease/Pathology**	Chronic heart failure exacerbations of chronic lung diseases, and infections	/	Pneumonia (37.7%), UTI (46.4%), and soft tissue infection (15.9%)	/	Cellulitis/skin and soft tissue infection (43%), UTI (17.9%), Rhabdomyolysis (15.9%)
**Morbidity**	/	COPD/CKD/diabetes/ Thromboembolism	Diabetes Cerebrovascular disease Dementia	Cardiovascular disorder Respiratory disorders Diabetes	No information

^1^: Significant difference; /: No information in study.

**Table 2:** Quality assessment of included studies.

	Jacobs 2007 [11]	Ang 2019 [9]	Tan 2025 [12]	Ayyildiz 2025 [10]	Ko 2026 [13]
Is there a well-defined question?	Y	Y	Y	Y	Y
Is there comprehensive description of alternatives?	Y	Y	N	Y	Y
Are all important and relevant costs and outcomes for each alternative identified?	Y	Y	Y	N	Y
Has clinical effectiveness been established?	N	N	N	N	N
Are costs and outcomes measured accurately?	Y	Y	Unclear	Y	Y
Are costs and outcomes valued credibly?	Y	Y	Unclear	Unclear	Y
Are costs and outcomes adjusted for differential timing?	NA	NA	NA	NA	NA
Is there an incremental analysis of costs and consequences?	Y	Y	N	N	Y
Were sensitivity analyses conducted to investigate uncertainty in estimates of cost or consequences?	N	N	N	N	Y
Do study results include all issues of concern to users?	N	Y	Y	N	Y
Are the results generalizable to the setting of interest in the review?	Y	Y	Y	Y	Y
Applicable criteria rated “Yes”, %	70%	80%	40%	40%	90%

**Table 3:** Clinical and Cost analysis of included studies.

	Jacobs 2007 [11]	Ang 2019 [9]	Tan 2025 [12]	Ayyildiz 2025 [10]	Ko 2026 [13]
**Main Outcomes**	Utilization rates of HH, general medical hospitalization and geriatric hospitalization days	Hospital admissions, ED visits and Inpatient stay, mortality, and cost	LoS, medical expenditure, ED visits, rehospitalization, patient satisfaction	Emergency service admission; hospitalization; mortality and survival; and hospital costs	LoS, 30-day readmission, 30-day ED reattendance, 30-day mortality, QoL, patient satisfaction
**Cost Data Source**	HMO bill	MOH	/	Electronic medical records	Patient billing data
**Currency**	USD	SGD	NTD	TRY	SGD
**Cost Classification**	Physician, nurses, physiotherapist, occupational therapist, social workers, drugs, management and logistics	Cost based on requirement outlined for each care service from MOH.	/	total bill amount	Courier services, patient and staff transportation, patient visits, medications, procedures, consumables, and tests conducted by external vendors
**Timeframe**	1y	180 days after hospital discharge	6m	/	Episode of care
**Statistical Analysis**	Linear regression	Difference-in-different approach, logistic regression	Logistic regression	NA	Stabilized inverse treatment probability weighting (IPTW)
**Covariate Adjustment**	/	Age, Charlson Comorbidity Index, dementia, ethnicity, functional status, gender, inpatient stay at baseline, level of care and ward class.	Multiple models adjusted for activity of daily living, cerebrovascular disease, and dementia		IPTW weights adjusted for age, gender, race, diagnosis at enrolment, Charlson comorbidity index, clinical frailty score, hospital admissions in last 12 months, health status and modified Barthel index
**Sensitivity Analysis**	No	No	No	No	Probabilistic sensitivity
**Clinical and Cost Outcome**	Surplus16,438 medical days and 6004 geriatric days Surplus costing 6.2 m. Following HH closure, a decline of 56% in HH days, cost-saving of 1.3 m. The increase trend is significant in hospitalization and recurrent admission.	Difference (TC vs. non-TC): LoS: −4.2 (−8.25–−0.14) * Hospital admissions:0.03 (−0.3–0.4) ED visits: 0.1 (−0.2–0.5) Readmission rate: non-TC vs. TC: 0.82 (0.44–1.54) Mortality: non-TC vs. TC: 0.69 (0.45–1.07). Cost saving S$2765/per patient. After offset of TC take-up: Cost savings S$1599/per patient. Total cost savings: Annual savings of S$1,119,300 (S$637,700 for patients and S$481,600 for the state) for 700 patients with shorter inpatient stay.	LoS: HaH: 7.5 (1.7) * Control: 10.9 (8.9) LoS (day), ≤7.5 aOR 0.53 (0.30–0.93) * ED visit <14 days HaH: 2 (2.9) Control: 5 (2.0) Rehospitalization < 14 days HaH: 7 (10.1) Control: 18 (7.3) Mortality HaH: 1 (1.5) Control: 16 (6.5) Satisfaction HaH: all Expenditure ≤ 29,209.9 0.18 (0.10–0.33) * Medical expenditure HaH: 29,209.9 (7839.6) * Control: 90,031.4 (153,854.5)	LoS in hospitalization: 7.12 (5.74) HHS: 8.33 (7.76) * non-HHS: 6.99 (5.47) LoS in ICU: 8.23 (13.70) HHS: 9.53 (14.60) * non-HHS: 8.08 (13.60) One-year survival: 5866 (68.2) HHS: 545 (62.9) * non-HHS: 5321 (68.9) Cost: 10,483 (21,534) HHS: 12,784 (26,308) * non-HHS: 10,225 (20,917)	Weighted difference (HaH vs. Control): LoS in hospital: −3.0 (−3.6, −2.4) * 30-day readmission: 1.2 (0.56, 2.58) 30-day ED reattendance: 1.9 (0.53, 6.53) Mortality: NA QoL: Change in EQ-5D: 0.03 (−0.06, 0.11) Change in EQ-VAS: 1.0 (−3.7, 5.6) Cost per episode: ED: −49 (−78, −21) * Ward: −3154 (−4136, −2173)*
**QoL or Satisfaction Indicators**		/	A 5-point Likert scale to assess patient and caregiver satisfaction, defining scores of 4 and 5 as “satisfied”	/	EQ-5D-5L, EQ-VAS

*: Significant difference; EQ-5D-5L: EuroQol 5-Dimension 5-Level Questionnaire; EQ-VAS: EuroQol Visual Analogue Scale; HaH: hospital-at-home; HH: home hospitalization; HHS: home healthcare services; HMO: Jerusalem Clalit Health Fund; LoS: length of stay; MOH: Ministry of Health; NTD: New Taiwan dollar; QoL: quality of life; SGD: Singapore dollar; TC: transitional care; TRY: Turkish lira; USD: United States dollar.

**Table 4:** Dominance ranking matrix.

Cost	No. of Studies	Health Benefit	Implication for Decisionmakers
+	1 (Ayyildiz 2025 [10])	−	Reject intervention
0		−	Reject intervention
+		0	Reject intervention
−		−	Unclear
0		0	Unclear
+		+	Unclear
−		0	Favor intervention
0		+	Favor intervention
−	4	+	Favor intervention

## Data Availability

No new data were created or analyzed in this study. Data sharing is not applicable to this article, as all data was obtained from published studies included in the systematic review.

## Abbreviations

The following abbreviations are used in this manuscript:

CKD	Chronic Kidney Disease
CHF	Congestive Heart Failure
COPD	Chronic Obstructive Pulmonary Disease
ED	Emergency Department
EQ-5D-5L	EuroQol 5-Dimension 5-Level Questionnaire
EQ-VAS	EuroQol Visual Analogue Scale
HaH	Hospital at Home
HMO	Jerusalem Clalit Health Fund
ICU	Intensive Care Unit
IUT	Intrauterine Transfusion
LoS	Length of Stay
MOH	Ministry of Health
NTD	New Taiwan Dollar
QoL	Quality of Life
SGD	Singapore Dollar
TRY	Turkish Lira
USD	United States Dollar
UTI	Urinary Tract Infection

## Appendix A

### Appendix A.1. Search Strategy in PubMed

18	#17 AND home [Title/Abstract]
17	#6 AND #12 AND #16, filter: English, Humans, from 2000–2026
16	#13 OR #14 OR #15
15	“mortality”[MeSH Terms] OR mortalit*
14	readmission OR “Patient Readmission”[Mesh]
13	“length of stay”[MeSH Terms] OR (length* AND stay) OR “length* of stay”
12	#7 OR #8 OR #9 OR #10 OR #11
11	(“economic*” AND “evaluation”) OR (“economic* evaluation”)
10	(incremental cost effectiveness ratio) OR (cost effectiveness ratio) OR ICER
9	cost effectiveness analysis
8	(cost* AND analysis) OR “cost* analysis”
7	“costs and cost analysis”[MeSH Terms] OR “Economics, Medical”[Mesh] OR “Economics, Nursing”[Mesh]
6	#1 OR #2 OR #3 OR #4 OR #5
5	medic* AND home
4	home AND (health service)
3	(hospital* OR “hospitalization”[MeSH Terms] OR “hospitals”[MeSH Terms] ) AND home
2	hospital-at-home[Title/Abstract]
1	“Home Care Services”[Mesh]

### Appendix A.2. Search Strategy in EMBASE

#23	#22 NOT ‘systematic review’/de NOT ‘meta analysis’/de NOT ‘practice guideline’/de NOT ‘questionnaire’/de NOT ‘editorial’/de NOT ‘letter’/de
#22	#21 AND [embase]/lim AND [2000–2026]/py AND [english]/lim AND [humans]/lim NOT [conference abstract]/lim
#21	#19 AND #20
#20	home:ti
#19	#7 AND #14 AND #18
#18	#15 OR #16 OR #17
#17	‘mortality’/exp OR mortalit*:ti,ab
#16	readmission*:ti,ab OR ‘patient readmission’/exp
#15	‘length of stay’/exp OR (length*:ti,ab AND stay:ti,ab) OR ‘length of stay’:ti,ab
#14	#8 OR #9 OR #10 OR #11 OR #12 OR #13
#13	economic*:ti,ab AND evaluation:ti,ab OR ‘economic evaluation’:ti,ab
#12	‘cost effectiveness ratio’:ti,ab OR icer:ti,ab
#11	cost:ti,ab AND effectiveness:ti,ab AND analysis:ti,ab
#10	‘cost effectiveness analysis’/exp OR ‘cost effectiveness analysis’:ti,ab
#9	cost*:ti,ab AND analysis:ti,ab
#8	‘costs and cost analysis’/exp OR ‘medical economics’/exp OR ‘nursing economics’/exp
#7	#1 OR #2 OR #5 OR #6
#6	medic*:ti,ab AND #4
#5	#3 AND #4
#4	‘home’/exp OR home:ti,ab
#3	‘health service’/exp OR (health:ti,ab AND service*:ti,ab) OR ‘health service’:ti,ab
#2	hospital*:ti,ab OR ‘hospitalization’/exp OR ‘hospitals’/exp
#1	‘home care’/exp OR ‘hospital at home’:ti,ab

### Appendix A.3. Search Strategy in Cochrane Library

#1	MeSH descriptor: [Home Care Services] explode all trees	3164
#2	hospital at home	20,012
#3	medic* OR (health service) OR hospital*	1,019,198
#4	MeSH descriptor: [Hospitalization] explode all trees	20,807
#5	(#3 OR #4) AND home	40,416
#6	(#1 OR #2 OR #3 OR #4 OR #5)	1,021,138
#7	MeSH descriptor: [Costs and Cost Analysis] explode all trees	16,690
#8	MeSH descriptor: [Economics, Medical] explode all trees	77
#9	MeSH descriptor: [Economics, Nursing] explode all trees	14
#10	cost* analysis	58,932
#11	cost effectiveness analysis	29,905
#12	#10 OR #11	58,932
#13	(incremental cost effectiveness ratio) OR (cost effectiveness ratio) OR ICER	11,036
#14	economic* evaluation	14,172
#15	(#7 OR #8 OR #9 OR #10 OR #11 OR #12 OR #13 OR #14 OR #15)	66,930
#16	MeSH descriptor: [Length of Stay] explode all trees	9669
#17	length* of stay	40,598
#18	MeSH descriptor: [Patient Readmission] explode all trees	1673
#19	readmission	10,294
#20	MeSH descriptor: [Mortality] explode all trees	18,583
#21	mortalit*	133,176
#22	(#16 OR #17 OR #18 OR #19 OR #20 OR #21)	168,754
#23	#6 AND #15 AND #22 with Cochrane Library publication date Between Jan 2000 and Jun 2026, in Cochrane Reviews, Trials	12,437
#24	“home”:ti	15,155
#25	#23 AND #24 with Cochrane Library publication date to Jun 2026, in Cochrane Reviews, Trials	277
